# Improving viral annotation with artificial intelligence

**DOI:** 10.1128/mbio.03206-23

**Published:** 2024-09-04

**Authors:** Zachary N. Flamholz, Charlotte Li, Libusha Kelly

**Affiliations:** 1 Department of Systems and Computational Biology, Albert Einstein College of Medicine, Bronx, New York, USA; 2 Department of Microbiology and Immunology, Albert Einstein College of Medicine, Bronx, New York, USA; The Ohio State University, Columbus, Ohio, USA

**Keywords:** artificial intelligence, protein language models, bacteriophage, computational biology

## Abstract

Viruses of bacteria, “phages,” are fundamental, poorly understood components of microbial community structure and function. Additionally, their dependence on hosts for replication positions phages as unique sensors of ecosystem features and environmental pressures. High-throughput sequencing approaches have begun to give us access to the diversity and range of phage populations in complex microbial community samples, and metagenomics is currently the primary tool with which we study phage populations. The study of phages by metagenomic sequencing, however, is fundamentally limited by viral diversity, which results in the vast majority of viral genomes and metagenome-annotated genomes lacking annotation. To harness bacteriophages for applications in human and environmental health and disease, we need new methods to organize and annotate viral sequence diversity. We recently demonstrated that methods that leverage self-supervised representation learning can supplement statistical sequence representations for remote viral protein homology detection in the ocean virome and propose that consideration of the functional content of viral sequences allows for the identification of similarity in otherwise sequence-diverse viruses and viral-like elements for biological discovery. In this review, we describe the potential and pitfalls of large language models for viral annotation. We describe the need for new approaches to annotate viral sequences in metagenomes, the fundamentals of what protein language models are and how one can use them for sequence annotation, the strengths and weaknesses of these models, and future directions toward developing better models for viral annotation more broadly.

## INTRODUCTION

Analyzing viral sequences depends on classifying those sequences into meaningful schema. For bacterial sequences, the foundational categories are defined by the taxonomic tree. Viral taxonomy, however, is complicated by the lack of a universal common ancestor for all viruses and thereby conserved marker genes (1), as well as the pervasive mosaicism observed in viral genomes (2). Due to the difficulty in classifying uncultivated virus genomes (UViGs) and their rapid increase in number uncovered exclusively in metagenomics, the International Committee on Taxonomy of Viruses has adapted previous taxonomic definitions to be focused on sequence parameters and metrics to be better aligned with virus discovery in the era of metagenomics (3, 4). While acknowledgment of the new reality has spurred efforts to better classify UViGs, the majority of new viruses remain unclassified (5–11).

This review is focused on the utility of protein language models (pLMs), a subset of machine- and deep-learning models, that are based on a transformation of amino acid sequences into vector representations that can be used for downstream classification, annotation, and modeling efforts. We direct readers interested in computationally distinguishing viral sequences from non-viral sequences to other tools and reviews, for example, see references 12 and 13 and also reference 14 for a recent review of bacteriophage identification tools in metagenomic sequencing data. The effort to annotate viral proteins is key because, having developed the physical and computational methods to access more viral sequences from the environment, we still need tools to understand the biology of viruses in ecosystems. This review is not a practical guide to the use of machine learning models. We direct readers to a recent review covering this topic (15). Finally, this review is not intended as a guide to best practices for using these models, primarily because protein language models are still a nascent tool for viral annotation; nonetheless, readers may find a review of general rules for protein sequence annotation with machine learning helpful (16).

We begin with an overview of how metagenomic data are used to annotate viral proteins and protein families, address current approaches and their limitations, and describe the potential for language models to improve viral protein sequence annotation. We provide a brief history of language models, describe their uses, their potential for biological discovery, and their pitfalls, and discuss current efforts to annotate protein sequences using protein language models. We end with a discussion of how to make protein language models more tailored to viral sequence annotation tasks and propose future goals for protein language models and other artificial intelligence (AI) approaches in annotation more broadly.

## STUDYING VIRUSES USING METAGENOMIC DATA

Given their host-dependent replication lifestyle, the study of phages has historically required a host experimental system. Their study has been in a laboratory setting, limiting the investigation to those phages suitable to such conditions. With the rise of high-throughput technologies, particularly genomic sequencing, the ability to assay microbial communities in their entirety and directly in their environment has launched a new era of microbiology. Metagenomics describes the study of community structure and function through the examination of bulk genetic material isolated from environmental samples and has been used to study ecosystems across the globe. The communities captured by metagenomic sequencing naturally contain phages, allowing, for the first time, the systematic characterization of phages in the wild. The new approach to environmental microbiology has ushered in an unprecedented study of phages in microbial communities, with hallmark catalog studies that describe the viral, including and predominantly phage, diversity in ecosystems. In the span of 5 years from 2016 to 2021, the number of UViGs increased 50-fold, with the pace of discovery unyielding (17).

Viral profiling studies, which have been performed from terrestrial (5) to aquatic (6) to host-associated microbiomes (7–9), constitute the foundation for environmental virology by answering questions of “who” on the way to answering questions of “how,” “where,” and “when.” Coupled with metagenomic sequencing, sophisticated computational methods have facilitated the investigation of UViGs in metagenomic data. It is necessary to label viral genetic material in bulk sequencing data, a difficult problem because viruses generally constitute a small fraction of the genetic material sequenced. A myriad of strategies and bioinformatic tools have been developed to identify viral sequences with both statistical and machine-learning methods that utilize sequence-based (18–23), gene-based (24, 25), and hybrid feature approaches (12, 13, 26, 27). Methodological advances have contributed to the proliferation of viral diversity uncovered in the age of metagenomics; however, confidently labeling viral sequences represents only the first step in analyzing UViGs.

## EXISTING CLASSIFICATION METHODS FOR UNCHARACTERIZED VIRAL SEQUENCES

**Fig 1:**
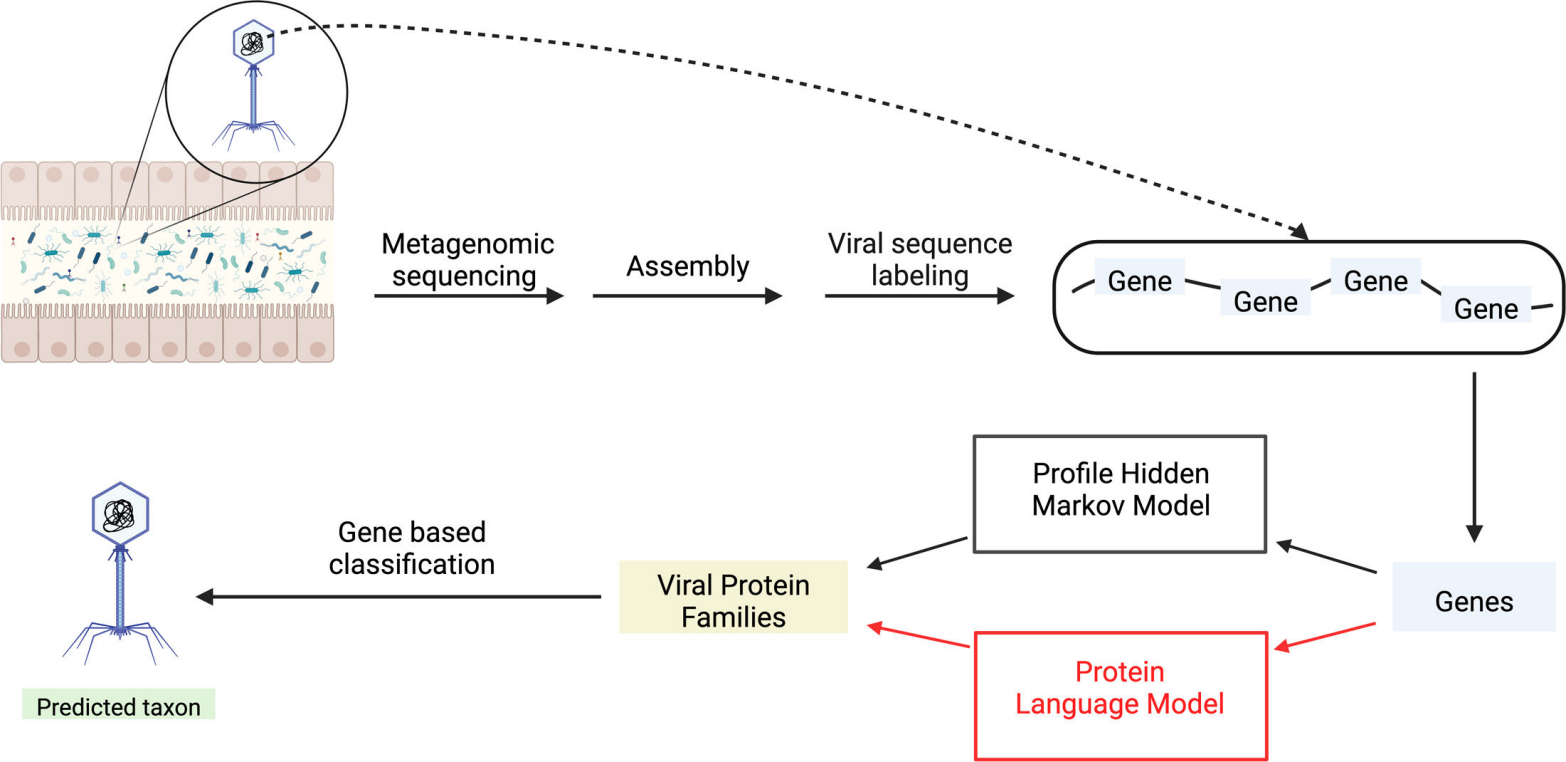
Annotation of viruses in metagenomes. Conceptual representation of a standard uncultivated viral genome taxonomic classification workflow.

Existing classification methods for UViGs rely primarily on gene-based features. Predicted protein sequences in UViGs are used as features for clustering-based (28–30) or machine learning-based (12, 31) classification. Gene features are defined as protein families that share sequence homology and are constructed by clustering a sequence-sequence distance matrix where the distance between two sequences is defined by a string distance function (32). A gene is determined to match a protein family if it statistically matches the sequence profile of the family, most often constructed as a profile hidden Markov model (pHMM) (33). There are a number of databases that catalog viral protein families (VPFs) and can be used to detect homology to known proteins (17, 34–36). However, the vast majority of predicted coding sequences do not share sequence homology with known protein sequences. Furthermore, even within databases of VPFs, most families lack functional annotation. Without VPF matches or annotation, the biological importance of the protein, and the genome as a whole, remain unresolved (Fig. 1).

Annotation by matching to databases of VPFs based on sequence homology suffers from two fundamental limitations. The first is the limited library of known and annotated viral protein sequences from which to construct probabilistic sequence models. Even with the massive increase in viral genomes and their aggregation in newly created viral sequence databases (37, 38), based on the rate of new sequence identification, databases have not reached protein sequence saturation (17, 36, 37), and many protein sequences in databases have only “unknown function” annotation. The second limitation of sequence models in viral protein homology detection is the rate at which viral proteins change, eventually diverging beyond recognition by traditional sequence homology while retaining functional homology (39). Unlike sequence, structure and function are maintained longer over evolutionary time due to biochemical and fitness constraints (40–42).

## THE NEED FOR NEW APPROACHES IN VIRAL ANNOTATION

To realize the biological insight and potential discoveries hidden in the terabytes of sequence data collected cataloging viruses, new approaches are necessary to interrogate UViGs and specifically their proteins. Viral protein sequences suffer from specific issues that make them difficult to annotate with current approaches. We know from published studies that state-of-the-art alignment-based approaches do not cover metagenomic viral sequence space. New functional annotation databases, such as the protein structure-focused AlphaFold, suffer from poor viral protein family structural representation (43). Sequence diversity and rapid evolution of viral sequences obscure evolutionary relationships. Viruses are required to retain specific functions, for example, head and packaging and other structural proteins (35). Some of these proteins can be very sequence diverse while carrying out the same function. By developing methods to identify very distant evolutionary relationships between proteins carrying out these functions, we may be able to better annotate viral sequences. As one example, bacterial double jelly roll viruses went undetected in environmental sequence databases, despite the identification and annotation of these types of viruses in eukaryotes, due to sequence diversity that rendered the bacterial sequences invisible to standard sequence homology identification approaches (44, 45). To emphasize this point, we are generating more and more viral data generated with less and less annotation; the problem of incorrect transfer annotation of functions based on classical approaches as databases grow is stark (46). Finally, it is difficult to characterize the biology of the myriad novel viruses being identified if we cannot annotate their genes. It is therefore particularly critical to explore novel approaches for annotation in the viral space.

## A BRIEF HISTORY OF REPRESENTATION LEARNING FOR PROTEIN SEQUENCE ANNOTATION

It has been known for decades that the function and structure of a protein are encoded in particular features of its sequence, such as domains, and that the presence of particular features in a sequence is predictive of the properties of a protein (47–49). Some important biological properties, such as the nuclear localization signal, were accurately predictable from sequence alone (50). In the vast expanse of function and structure, however, the number of predictable properties was small, and even more so for proteins found outside of eukaryotes. Predictive features needed to be curated with defined rules and were based on accumulated biological knowledge, or in the parlance of machine learning, the features were engineered. For microbial proteins, such investigation is impossible at the scale that would be needed to experimentally identify predictive features.

In recent years, while metagenomics was exposing the universe of microbial diversity, advances in machine learning, specifically natural language processing (NLP), displayed the power of learned representations for capturing meaning contained in the sequence of symbols. Representation learning is a subfield of machine learning concerned with learning optimal representations of inputs for other learning tasks (51). Representations can be learned implicitly, as is the case when training is done directly for a final task or can be done explicitly with a specific training objective designed to shape the representations learned (51).

Representations can be trained in a supervised or unsupervised manner; however, the substantial gain of representations in the era of deep learning has been the ability to train unsupervised, specifically self supervised, over vast amounts of data (52). In NLP, representation learning was transformed from word count-based to word context-based, with inspiration from the distributional hypothesis in linguistics articulated by linguist Dr. Zellig Harris, who posited that words that appear in similar contexts have similar meanings (53). The first training architecture to implement the context-based approach, word2vec (54), revolutionized NLP, and newer architectures have built off this fundamental concept. It did not take long for the obvious parallels of written text to biological sequences to motivate the testing of NLP models, architectures, and objectives for training representations of protein sequences (55).

Learned representations, when represented as dense, real-number vectors, are called embeddings. For proteins, a natural vocabulary to model is the set of amino acids. In the simplest parallel to natural language modeling, the objective for training representations is to predict the amino acid at a position in a protein sequence given the sequence context. Such models have been termed pLMs and transform a protein sequence into a high-dimensional vector for each amino acid in the sequence. Embeddings can be used as inputs for other learning tasks, usually supervised, in a process of transfer learning. Embeddings can also be interrogated themselves to understand what a model has captured in the representation, usually with unsupervised clustering methods. With newer architectures and larger training data sets used to train pLMs, the models captured physico-chemical properties of amino acids and protein sequences in their dense representations (56–61). These self-supervised models, trained on millions of protein sequences, could resolve protein structural and functional information from only sequence input (56–61). Additionally, the desire for more training data led to the inclusion of microbial proteins into training corpora from databases like Uniprot and Protein Data Bank and even uncharacterized proteins predicted from uncultivated genomes in metagenomic sequence data (59, 60). Such models, having been trained on a vast collection of microbial proteins, could encode features related to the function of the protein in a protein representation. With respect to virus annotation, analyzing proteins identified in never-before-seen-virus genomes with pretrained pLMs could help bridge the current gap with existing sequence homology approaches (Fig. 1).

## A PRACTICAL INTRODUCTION TO LANGUAGE MODELS, WITH A FOCUS ON PROTEIN LANGUAGE MODELS

Large language models (LLMs) are AI models that are trained on a large amount of input in an unsupervised or self-supervised manner. pLMs are a specific NLP algorithm that uses AI to categorize proteins in a conceptually similar way to classical multiple sequence alignments and pHMMs (62). Specifically, pLMs are machine-learning models that are trained on data sets of amino acid (AA) sequences (62). When given a new AA sequence as input, these models can learn and predict useful protein features, for example, the secondary structures of proteins, their biological functions, and their residue-residue contacts (58). pLMs recognize patterns in text and can be applied to annotating viral proteins due to representational similarities between the human language, which utilizes the alphabet, and proteins, which utilize AA sequences (63). Similar to how words have different meanings depending on their context in a sentence, AAs can behave differently depending on their interactions with neighboring, or distant-in-sequence, AAs. Just as NLP algorithms create numerical representations of sentences and are able to capture the context of words in the human language, NLP-vectorized AA sequences can be used to predict the structure and function of proteins in an organism (55).

**Fig 2:**
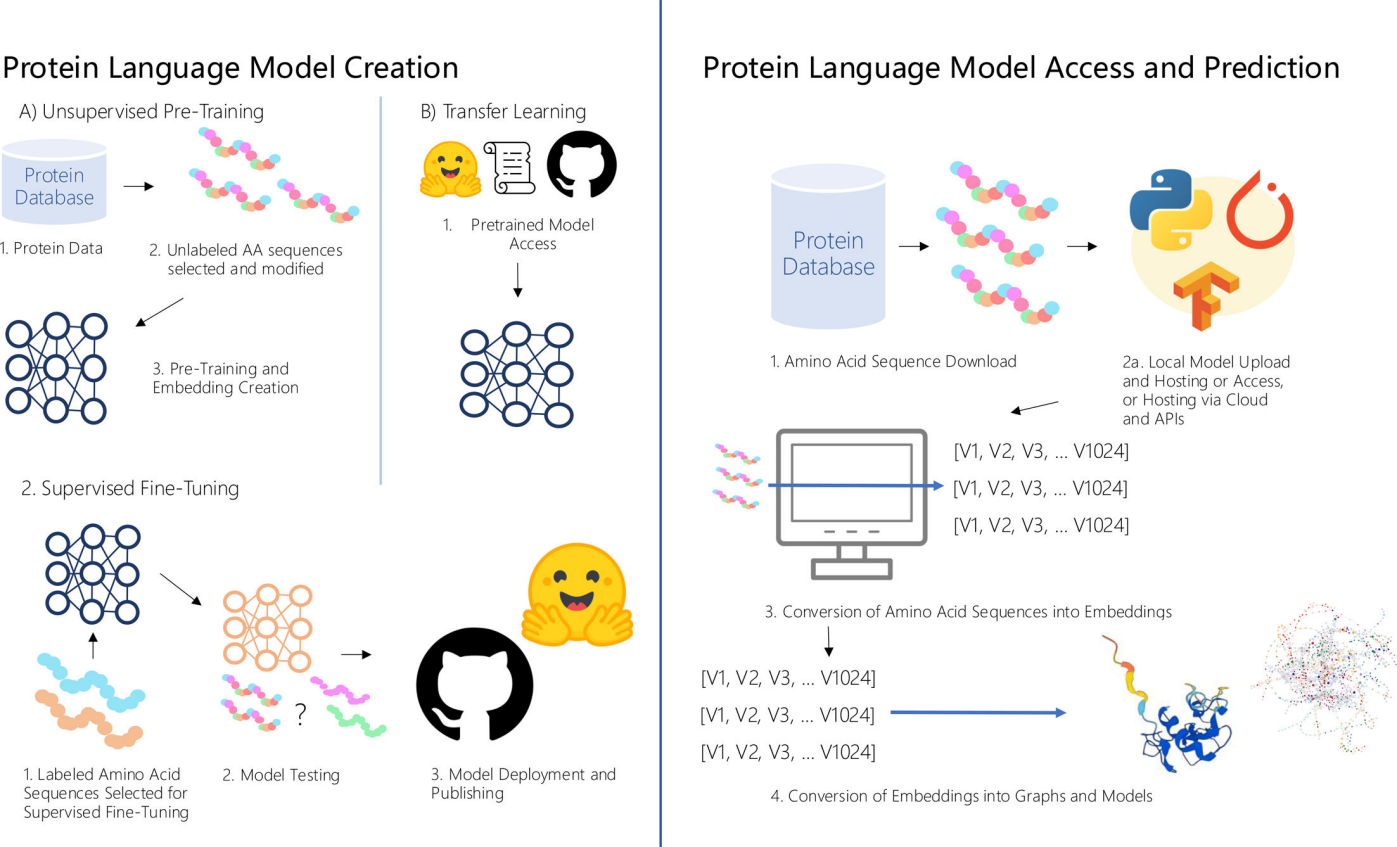
Overview of pLM creation, deployment, and utilization. (Top left) (**A**) Protein language model creation. (1) Unsupervised pretraining. Protein data are downloaded and extracted from online databases of AA sequences. (2) AA sequences are modified based on the objective of the pLM; for example, the AA sequences can be transformed into vectors. (3) AAs are separated into groups and inputted into the machine-learning models. The models determine weights and attention after recognizing patterns in the amino acid data. (**B**) Transfer learning. Code for a pretrained model, and/or the predetermined weights and attention of a pretrained model, are downloaded from repositories such as Github or Huggingface. (Bottom left) (1) Supervised fine-tuning. Labeled AA sequences are input into the pretrained machine-learning models. The machine-learning models refine their weights and attention and develop more task-specific outputs. (2) Models are tested using held-out sequences with known labels or structures. Known labels and structures are then compared to the model’s predicted labels and structures to assess accuracy. (3) Completed models are published on HuggingFace (https://huggingface.co/), Github (https://github.com/), or other platforms, where they can be downloaded or hosted. Right panel: Protein language model access and utilization. (1) Protein data are downloaded and extracted from the online AA sequence databases. (2) Machine-learning models are accessed via (i) download or (ii) cloud and application programming interfaces (APIs). If downloaded, the model is run locally using the computer’s resources on programs such as Python. (3) If accessed via the cloud or APIs, the model is run on an external device, and the outputs are sent from this external device to the user’s local device. (4) The model converts AA sequences into embeddings. The embeddings are converted into graphs and models through decoding, where trained decoders turn sequence embeddings back into sequence data; dimensionality reduction, where the multidimensional embedding vectors are reduced to plottable coordinates; clustering algorithms, where similarities in the embedding data are found and represented graphically, for example. Images from pngtree.com (computer image), Huggingface.co, CleanPNG (python logo), https://alphafold.ebi.ac.uk/entry/P79682 (protein structure), and Github.

Three key aspects of protein language model design and access relevant to viral protein annotation are (i) what data sets the models are trained on, (ii) model architecture, and (iii) model training objectives (Fig. 2).

### Model training

Protein databases, such as GenBank (https://www.ncbi.nlm.nih.gov/genbank/) and Uniprot (https://www.uniprot.org/), contain billions of protein sequences clustered using multiple sequence alignments and HMMs. Amino acid sequences from these databases are directly inputted into models. Depending on the model, these sequences can also be altered before they are inputted into the model by being tokenized or split into smaller representative sequences. Then sequences are converted into numerical representations. Big fantastic database (BFD) is one of the most commonly utilized databases for pLM training. It contains 2.5 billion clustered proteins from several smaller databases (https://bfd.mmseqs.com/). BFD is used in AlphaFold for protein structure prediction (43), for protein-level assembly of metagenomic sequences (64), and for clustering large protein data sets (65). BFD includes metagenomic sequences and thus includes viruses sequenced from environmental samples, making it a good foundational database for using pLMs to annotate viral protein families (66).

### Model architectures

The architecture of a model, or its structure, affects its training time, memory usage, accessibility, and accuracy. The choice of training architecture, as well as training data, also changes the model’s bias, creating tradeoffs in what parts of the data the model is able to focus on while learning. Major examples of older language model architectures include n-gram, skip-gram, and bag-of-words language models. These language models are all statistical language models, which apply statistical estimation techniques to determining patterns in languages (67).

N-gram language models are language models that determine the probability of a word being used in a sentence depending on the n-1 words preceding it (68). Skip-gram language models use a current word to predict its context given surrounding words. Conversely, bag-of-words language models use the context provided by surrounding words to predict a current word (69).

Word2Vec is one of the earliest examples of a successful NLP utilizing a neural network to produce embeddings and uses several of the older model architectures listed above. Specifically, Word2Vec consists of (i) a skip-gram model and (ii) a continuous bag-of-words model (69). ProtVec is a pLM based on the model architecture of Word2Vec. However, instead of being trained on words and sentences, ProtVec was trained on AA sequences from SwissProt, which were broken up into “words” using an n-gram model of size 3 (56).

Major examples of current pLM architectures include recurrent neural networks, long short-term memory models, encoder-decoder models (which include transformer models), large language models, and small-scale language models.

Recurrent neural networks (RNNs) are a form of neural network where the model passes outputs from previous states into new states. The recurrence allows these models to process sequences of indefinite length. Consequently, RNNs are good for processing AA sequences, which can have variable lengths from tens to thousands of amino acids. However, RNNs cannot learn patterns in the AA sequence data as deeply (70).

Long short-term memory models (LSTMs) are RNNs with an additional memory cell that stores information from previous states. These models are able to recognize global patterns in sequence data, as well as patterns between AAs that are farther apart in the sequence. LSTMs were proposed, in the background of random forest algorithms, multiple sequence alignments, and logistic regression, for determining the function of proteins. As an example protocol for predicting functional classes, protein data from the UniProt database was extracted and sorted based on clasees of proteins. The LSTMs were trained on these different classes. LSTMs were able to capture more distant relationships between AA data and protein functions but may struggle when run on larger databases where functional classes are poorly represented (70, 71).

Convolutional neural networks (CNNs) are multilayered neural networks. The input data are processed by one or more convolution layers. CNNs require significantly less memory and computational power to process data than transformers do. Moreover, CNNs are able to study longer AA sequences, while transformers have to limit the protein sequence length during pretraining and prediction (72).

Encoder and decoder models can be any form of model, including an LSTM, RNN, or transformer model. In an encoder-decoder architecture, an encoder model will accept AA sequences as input and create a contextualized representation of the data. Then, a decoder will take the data from the encoder model and create an output dependent on the objective of the model (68). The AnnoPRO model uses encoding-decoding to annotate the functions of proteins and overcome the long-tail phenomenon, where head protein families are far better annotated by pLMs than tail protein families. This issue is caused by having too many unannotated families in a protein database, which leads to the domination of proteins in the “head label levels” category (73).

Transformer models are a specific form of encoder-decoder models. These models are more efficient than their LSTM encoder-decoder counterparts because they use attention mechanisms, which are set weights that determine what parts of a sequence a model should pay attention to. As a result, the entire sequence is not required to make sense of a given part of the data. Moreover, different attention mechanisms for the encoder and decoder allow the two models to be trained in parallel (74). Encoder and decoder models can be used separately depending on the task. ProstT5 is a transformer model that uses an encoder model to generate embeddings from an AA or sequence of structural tokens, called 3Di states. The model then uses a decoder on these embeddings to convert from AA to 3Di and vice versa (75).

Smaller-scale protein language models (SS-pLMs) are also used to annotate proteins. These models are more accessible than LLMs in that they are trained on smaller sequence databases and consequently have lighter computational loads. In protein generation tasks, decreasing the number of protein sequences in the fine-tuning step led to less diversity in generated sequences and generated protein sequences deviated more from the target enzyme family under study. However, when SS-pLMs were trained on protein data sets specific to a given protein family, their ability to generate functionally effective protein sequences, such as protein sequences with increased binding affinity, increases (76).

### Training objectives and model uses

The training objective of a pLM determines its structure, training, and output. The pLMs referenced below are examples of different model architectures and usages.

Convolutional autoencoding representations of proteins (CARP) models are protein language models based on CNNs rather than transformers. Because the CNN-based CARP models process ranges of AA sequences at a time, they capture positional information between relatively close AAs. Moreover, CARP models are able to pretrain on longer sequences and process longer sequences than transformers due to their efficiency (72).

Foldseek is a search engine that finds structurally similar matches to an input AA sequence. Foldseek uses a vector-quantized autoencoder model (VQ-VAE) to turn the AA sequence into a sequence representing the predicted structure of the protein. The search engine utilizes a structural “alphabet” of 3Di states, which represent the different interactions between protein residues. Then, Foldseek compares this structural sequence to other structural sequences in protein databases using sequential alignment algorithms such as the Smith-Waterman algorithm. Foldseek uses the VQ-VAE, a form of encoder-decoder model, to predict the structural sequence of the AA sequence from a training data set of maximally evolutionarily conserved 3Di states (77). As an example of a protein-annotation task, Foldseek was used to validate the discovery of replication proteins in cyanobacteria (78).

ProstT5 is a transformer model that converts between 3Di tokens and AA sequences. ProstT5 was fine-tuned from ProtT5, an older transformer model that was trained with three billion parameters on Uniref50 and BFD to determine the structure of proteins. Embeddings from the encoder were used to train the second half of the model (59, 75). A new protein alignment approach based on pLMs called embedding-based alignment was proposed. In this approach, proteins are aligned and annotated using similarity matrices between their embeddings (79).

ProGen is a conditional transformer model that generates protein sequences. ProGen was trained on a set of AA sequences with associated “conditioning tags,” for example, labels that assign a function to a particular AA sequence. Conditioning tags are labels assigned by databases and specify the molecular function, biological process, taxonomy, etc. of a given protein. ProGen will take in a set of these conditioning tags as input and then build a protein satisfying those properties (80).

ESMFold is a large language transformer model that predicts structure from sequence. This model was trained on 15 billion parameters, as opposed to the millions of parameters that older pLMs were trained on (76). ESMFold was used to generate the ESM Metagenomic Atlas database, which contains over 600 million proteins and their structures (60).

AnnoPRO is an encoder-decoder model that is pretrained on both feature similarity-based images (ProMAP) and vectors capturing global protein similarity (ProSIM). It uses a seven-channel CNN and a deep neural network of five layers to encode proteins. Then, AnnoPRO decodes and annotates these protein features using an LSTM (73).

pLMs can also be fine-tuned for domain-specific learning objectives. For example, the genomic language model (gLM) uses metagenomic scaffolds to fine-tune a pretrained pLM, ESM2 (60), to consider the genomic context of a protein in its representations (81). The inclusion of genomic context information was shown to enhance downstream predictive power for tasks in microbiology like taxonomy prediction and enzyme function classification.

### Model availability

The scripts for generating protein language models are generally provided as part of their publication. After the pLMs are trained, they can be downloaded, deployed and run from different online platforms that host these models. pLMs can be run locally (directly on the main device) or virtually, where another device runs the machine learning model and then sends the results to the main device. If run locally, the protein language models are downloaded from repositories such as Github (http://github.com/) or Huggingface (http://huggingface.co/), which store their code. The code is then run on the main device using platforms such as Python or Visual Studio Code. If run virtually, the script for the machine learning model is uploaded onto another computer. These computers, or virtual machines (VMs) can be accessed through Google Colaboratory (https://colab.research.google.com/), or cloud computing companies. The basic RAM requirement for machine learning ranges from 8 GB to 16 GB. The requirements for RAM, CPU and GPUs vary, however, depending on the architecture of the models being run, the duration over which they are being run and the size of the input data sets.

## A USE CASE: ANNOTATING PROKARYOTIC VIRAL PROTEIN FAMILIES USING A PRETRAINED PLM

A major limitation of pLMs is that their training requires vast computational resources. As an example, the ProtTrans model was trained on supercomputers at the Oak Ridge National Laboratory (59). However, available pLMs can be used to extend viral protein sequence family annotation. We used pretrained pLMs and available viral protein family databases to demonstrate that the protein language models can capture viral protein function in metagenomic data sets (66). Specifically, we embedded our sequences into to vectors using the Transformer_BFD pLM from the ProtTrans project (59) using the DeepChainBio/BioTransformers Python package (https://github.com/DeepChainBio/bio-transformers) and developed a classifier by training and testing with the PHROG data set of viral protein families (35). When applied to global ocean virome data, our classifier expanded the annotated fraction of viral protein families and discovered novel mobile genetic elements and viral capsid proteins that were widespread in the global oceans. We note that due to conserved evolutionary relationships between eukaryotic and prokaryotic viral proteins, our models can identify proteins from viruses that infect across all domains of life despite not being tailored to eukaryotic viruses (35). However, future work seeking to identify all viruses in an environmental sample, for example, will likely require specific training to identify proteins from viruses from each domain of life.

## FUTURE DIRECTIONS

### Findable, accessible, interoperable, and reusable data principles in protein annotation

Our studies, and others exploring the world of viruses in our environment, benefited from many existing computational methods and data resources. For future work to take the most advantage of the vast universe of protein data to learn about the world around us, we briefly discuss the power of adoption of findable, accessible, interoperable, and reusable data principles (81) in the area of bioinformatics (82) to support the development of research products that can be leveraged by the larger scientific community.

Metagenomic data analysis and sequence homology searching for viruses benefit from diverse developers and methods, including, but not limited to, the bioBakery suite (83), tools from the CyVerse Discovery Environment (84), and interfaces from the MPI Bioinformatics Toolkit (85), PHROGs (35), ProtTrans (59), EFAM (36), geNomad (12), and VIBRANT (25), all of which are supported by thorough documentation and maintained by a community of developers. Scientific workflow software solutions, such as nextflow and the nf-core/mag pipeline (86), have reduced the difficulty of tool implementation and integration, allowing for complex pipeline design, scale, and publication. In the field of studying the microbiome, efforts to analyze sequence data homogeneously and harmonize metadata have begun to appear in the community, especially for human-associated microbiome studies (87, 88). With our viral annotation method, we produced a no-code graphic user interface through a Google Colab notebook to enable researchers with limited experience with computational biology to apply AI models to their own data (66). The delivery of usable and intuitive computational methods, tools, and data resources to domain scientists has primed the field for exciting discoveries in the years ahead and must continue to be supported.

### pLMs for virus-specific objectives and biological questions

Limitations in the specificity of currently available models to questions in viral biology may restrict viral research community adoption of these tools. Here, we propose some next steps to develop models tailored to open questions in viral biology. One could train virus-specific pLMs, potentially by utilizing large sequences of viral and mobile elements such as IMG/VR (17). Viral genes with similar functions often co-localize in viral genomes (89), and this feature could enable the use of genomic neighborhoods or “context” of particular viral protein families to improve annotations in viral-focused models conceptually similar to the gLM (90). We found that even in diverse metagenomic sequences, high-level functions are conserved in the neighborhoods of related viral elements (66). An area of interest for viral ecology generally and efforts to use viruses in a clinical context, such as phage therapy, is the ability to predict the host range of a phage; interactions between phages and hosts can leave signatures in phage genomes (91) that may be identifiable by protein- or DNA-based language models. pLMs could enable better insight into evolutionary relationships between phages and related mobile elements that contain phage genes, including, but not limited to, phage defense systems (92), phage-inducible chromosomal islands (93, 94), and tycheposons (95). Models could also be tailored to different environments. For example, our classifier, which was tuned for performance on marine metagenomes (66), could be re-trained and tested on other environments, such as the human gut. We note that this review has focused on bacteriophages; pLMs specific to eukaryotic virus annotation tasks could improve the discovery of eukaryotic viruses in environments. As an example, sewage has been shown to contain abundant eukaryotic viruses, including novel and abundant viral families associated with human waste (96).

## THE VIRAL RESEARCH COMMUNITY AND PLMS

We want to end by noting that the rapid progress in the development and deployment of LLMs and pLMs to the broader scientific community has also come with reasonable skepticism. The community adoption of tools described here could be framed as akin to the first deployment of BLAST, today the most commonly used tool to detect sequence similarity between a query sequence of interest and targets from huge biological databases, by the National Center for Biotechnology Information, in 1989 and published in 1990 (97). The tools described here may provide similarly new leaps forward in our ability to annotate the vast wealth of sequences in viruses and to understand novel aspects of viral biology. One can imagine the next generation of tools where representation-based searching and analysis are done on model-learned representations of protein sequences. However, we need to keep our eyes on interpretability, validation, and verification of predictions made if these models are to be adopted widely.
